# G-Quadruplex Conformational Switching for miR-155-3p Detection Using a Ligand-Based Fluorescence Approach

**DOI:** 10.3390/biom15030410

**Published:** 2025-03-13

**Authors:** Pedro Lourenço, Carla Cruz

**Affiliations:** 1 RISE-Health, Department of Medical Sciences, Faculty of Health Sciences, University of Beira Interior, Av. Infante D. Henrique, 6200-506 Covilhã, Portugal; pedro.afonso.amaro.lourenco@ubi.pt; 2 Department of Chemistry, University of Beira Interior, Rua Marquês d’Ávila e Bolama, 6201-001 Covilhã, Portugal

**Keywords:** miR-155-3p, molecular beacon, G-quadruplex, lung cancer

## Abstract

MicroRNA-155-3p (miR-155-3p) is an important biomarker in various pathological conditions, including cancer, making the development of sensitive and specific detection methods crucial. Here, we present a molecular beacon (MB-G4) that underwent a conformational switch upon hybridization with miR-155-3p, enabling the formation of a G-quadruplex (G4) structure. This G4 was recognized by the fluorogenic ligand N-methyl mesoporphyrin IX (NMM), producing a fluorescence signal proportional to the target concentration, making it a new detection method. The conformational dynamics of MB-G4 were characterized through circular dichroism (CD) spectroscopy and native polyacrylamide gel electrophoresis (PAGE), confirming the transition from a hairpin structure to an RNA–DNA hybrid duplex that facilitated G4 formation. The optimization of the experimental conditions, including the potassium chloride (KCl) and NMM concentrations, ensured selective detection with minimal background signal. The detection limit (LOD) was determined to be 10.85 nM, using a linear fluorescence response curve, and the specificity studies demonstrated a clear distinction between miR-155-3p and miR-155-5p. Furthermore, MB-G4 was studied with total RNA extracted from the lung cancer cell line A549 to evaluate its detection in a more complex environment and was able to detect its target, validating its potential for biological sample analysis.

## 1. Introduction

Lung cancer is one of the most prevalent cancers globally and a major cause of cancer-related deaths, with high rates of morbidity and mortality [1], with non-small cell lung cancer (NSCLC) representing around 85% of all lung cancers and mainly consisting of two histological subtypes—adenocarcinoma and squamous cell carcinoma [2,3]. Significant progress has been made in the diagnosis and treatment of lung cancer through molecular biology, particularly with the identification of novel biomarkers [4], and among these, microRNAs (miRNAs) have emerged as key regulators.

miRNAs are small, non-coding RNA molecules that regulate gene expression at the post-transcriptional level [5]. These function by binding to complementary sequences in target messenger RNAs (mRNAs), where they can either inhibit translation or promote mRNA degradation, regulating a wide range of cellular processes [6,7], such as development, differentiation, immune responses, and homeostasis [8,9,10]. Alterations in miRNA expression are closely associated with the development of various diseases, including cancer [11,12], positioning miRNAs as both promising biomarkers and effective targets for therapeutic interventions [13].

miR-155 has emerged as a particularly important regulator due to its involvement in a wide range of biological and pathological contexts [14], since it has been shown to play a role in regulating immune responses and inflammatory signaling, and its overexpression has been associated with various cancers, including lung cancer [15,16]. The ability to detect miR-155 with specificity and sensitivity is therefore crucial for advancing diagnostic strategies and gaining a deeper understanding of its functional role in disease progression [17]. Currently, there are various methods to detect miRNAs, such as quantitative reverse transcription PCR (qRT-PCR), microarrays, and next-generation sequencing (NGS). However, these often prove to be time-consuming and expensive [18,19].

DNA-based biosensors have gained significant attention due to their versatility and accuracy in detecting nucleic acids, with an example being the use of DNA hairloop structures, which can undergo conformational changes in response to molecular interactions. These hairloop structures can be engineered to include guanine-rich sequences that, under specific conditions, form G-quadruplex (G4) structures, secondary DNA configurations stabilized by guanine tetrads connected through Hoogsteen hydrogen bonds [20]. Known for their structural complexity, these G4 structures play crucial roles in biological processes, such as transcriptional regulation and genome stability, and have been applied in a variety of fields [21].

One key feature of G4 structures is their ability to be recognized by specific ligands [22]. Some of these ligands undergo fluorescence enhancement when binding to G4 structures, allowing for their detection and generating measurable signal changes, such as deviations in the absorbance or emission spectra [23]. This interaction offers an indirect approach for detecting G4 formation and the molecular events that trigger it.

**Figure 1:**
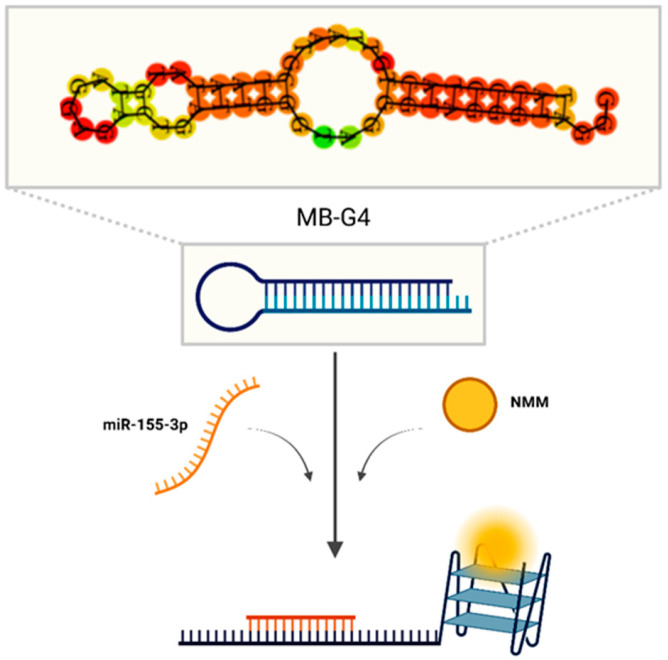
Prediction of the MB-G4 structure using RNAfold software (version 2.6.3) and schematic illustration of the conformational switch upon target hybridization, releasing the G-rich sequence and allowing G4 formation and subsequent recognition by NMM.

This study aims to develop a detection method of miR-155-3p through the structural dynamics of DNA hairloop transitions and G4 formation and further detection by N-Methylmesoporphyrin IX (NMM). By looking at the unique interplay between a hairloop DNA structure and a miRNA interaction, this work shows the potential of DNA-based biosensors as a tool for molecular diagnostics. Figure 1 represents the proposed method.

## 2. Materials and Methods

### 2.1. Oligonucleotide Sequences

The oligonucleotides used are presented in Table 1 and were purchased from Eurogentec (Seraing, Belgium). Oligonucleotide concentration was checked using the absorbance at 260 nm (NanoDrop™ 2000c, Thermo Scientific™, Waltham, MA, USA) and the molar extinction coefficients (ε) provided by the manufacturers (Table 1). Oligonucleotide stock solutions were prepared using Milli-Q and RNAse-free water and stored at −20 °C. Where necessary, oligonucleotide annealing was performed by heating the sample to 95 °C for 5 min, followed by fast cooling on ice for 10 min. All chemicals (Table S1) were purchased from Sigma-Aldrich (Waltham, MA, USA) and MedChemExpress (Monmouth Junction, NJ, USA).

### 2.2. Fluorescence Studies

High-precision quartz Suprasil cuvettes with light paths of 3 × 3 mm (Ref. 105–251–15–40, Hellma Analytics, Müllheim, Germany) were employed for fluorescence studies, performed using a FluoroMax4 spectrofluorometer (Horiba, Kyoto, Japan). Fluorescence spectra were recorded in the 550–750 nm wavelength range, with an excitation wavelength of 399 nm. All spectra were scanned with the emission and excitation slits set to 5 nm and averaged over three scans. All data were analyzed using OriginPro 2024 software (v.10.1.0.178) (OriginLab Corporation, Northampton, MA, USA).

The experimental settings were optimized prior to data collection (details in Supplementary Materials).

After optimizing the experimental conditions, MB-G4 was annealed following the previously established protocol and prepared in a mixture containing 50 nM MB-G4, 25 nM miR-155-3p, and 1 μM NMM in a buffer comprising 5 mM KCl, 10 mM LiCaco, and 10 mM MgCl_2_. The mixture was then incubated at 50 °C for 30 min to facilitate hybridization.

#### 2.2.1. Limit of Detection

For detection validation, after optimizing the experimental conditions, 50 nM of MB-G4 was annealed in a 10 mM Licaco buffer. From this solution, a working mix containing 50 nM MB-G4, 1 µM NMM, 5 mM KCl, 10 mM Licaco, and 10 mM MgCl_2_ was prepared. To determine the limit of detection (LOD) of miR-155-3p, varying concentrations of the synthetic miR-155-3p sequence were prepared in this mix and then heated at 50 °C for 30 min.

The LOD was calculated based on the standard deviation (σ) of the blank signal and the slope of the calibration line and may be expressed as follows:

(1) $$LOD=\frac{3\sigma }{Slope}$$

The specificity of the MB-G4 designed was evaluated using the nonspecific miR-155-5p sequence as control and with the same concentrations and hybridization conditions.

#### 2.2.2. Total RNA Detection

To validate the application of the MB-G4 in detecting miR-155-3p in complex biological samples, experiments were performed using total RNA extracted from the A549 cell line (refer to Section 2.5 for cell culture conditions and RNA extraction protocols). Under the optimized experimental parameters, 250 ng of total RNA was added to the annealed MB-G4. This step enhanced the assay’s sensitivity for detecting miR-155-3p in a complex RNA environment. Control samples, containing only MB-G4 and 250 ng of total RNA without the target miRNA, were processed in parallel.

### 2.3. Circular Dichroism (CD) Spectroscopy

The circular dichroism (CD) spectra were recorded using a Jasco J-815 spectropolarimeter (Jasco, Easton, MD, USA) equipped with a Peltier temperature controller (model CDF-426S/15) set to 20 °C. Measurements were performed in the 200–320 nm range using a quartz cuvette with a 10 mm path length (Ref. 110-1-40, Hellma Analytics, Müllheim, Germany). The scanning parameters included a speed of 100 nm/min, a bandwidth of 1 nm, and an integration time of 1 s per point, with data averaging over four accumulations.

For all the CD experiments, the MB-G4 sequence was prepared at a concentration of 5 μM and annealed in 10 mM Licaco buffer. For miR-155-3p, a concentration of 2.5 μM was used.

The optimization experiments involved preparing solutions containing the same concentrations of MB-G4 and miR-155-3p, along with increasing amounts of KCl.

For the characterization studies, solutions were prepared containing MB-G4 and miR-155-3p at specified concentrations in a buffer comprising 5 mM KCl, 10 mM Licaco, and 10 mM MgCl_2_. All data were analyzed using the OriginPro 2024 software (OriginLab Corporation, Northampton, MA, USA).

### 2.4. Non-Denaturing Polyacrylamide Gel Electrophoresis (PAGE) Analysis

Polyacrylamide gel electrophoresis (PAGE) was used to confirm the formation of a G4 structure by the MB-G4, both in the presence of KCl and the hybridized target. A non-denaturing 20% polyacrylamide gel was prepared and assembled in a Mini-Protean II vertical electrophoretic cell (Bio-Rad, Hercules, CA, USA) connected to a PowerPac™ power supply (Bio-Rad, Hercules, CA, USA). Prior to sample loading, the gel was pre-run at 90 V for 30 min at 4 °C.

The oligonucleotide samples were prepared at a final concentration of 1 µM in Milli-Q water, heated to 90 °C for 5 min, and subsequently incubated at 50 °C for 30 min in a buffer containing 10 mM lithium cacodylate. For the G4 formation studies involving miR-155-3p, the buffer additionally contained 5 mM KCl. Following incubation, 10 µL of 60% sucrose solution was added to the samples before loading them onto the gel.

Oligonucleotide markers, consisting of sequences with lengths of 9, 15, 21, 30, 60, and 90 nucleotides, were loaded in parallel to the samples. Electrophoresis was performed at 120 V for 150 min at 4 °C. Afterward, the gel was incubated with NMM for 30 min under continuous gentle agitation to detect parallel G4 structures. The gel was then washed and stained with 1× SYBR Gold for 15 min with gentle agitation. Visualization of the results was conducted using a ChemiDoc™ XRS system (Bio-Rad, Hercules, CA, USA).

### 2.5. Cell Culture and Total RNA Extraction

A549 cells were cultured in Ham’s F12 medium (Gibco, Waltham, MA, USA), supplemented with 10% fetal bovine serum (FBS) (Gibco, Waltham, MA, USA) and 1% streptomycin–penicillin (SP) (Gibco, Waltham, MA, USA) antibiotics, in a humidified incubator at 37 °C with 5% CO_2_. When the cells reached 80–90% confluence, they were collected. The total RNA was extracted and purified using the miRNeasy Mini Kit (Qiagen, Hilden, Germany), and its concentration was determined using a NanoPhotometer (IMPLEN, Munich, Germany).

## 3. Results

The goal of this study was to develop a rapid, enzyme-free detection method for miR-155-3p using a DNA hairloop capable of forming a G4 structure upon hybridization with the target miRNA. To achieve this, the experimental conditions with miR155-3p were systematically optimized, including salt concentration, ligand concentration, hybridization temperature, and incubation time, and the results are detailed in Supplementary Materials.

### 3.1. Assessment of G4 Formation by MB-G4

**Figure 2:**
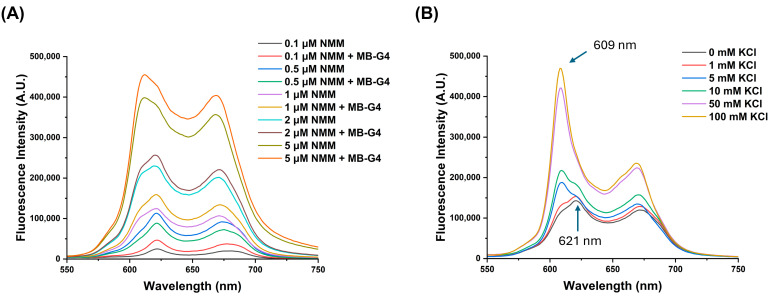
Fluorescence-based analysis of G4 formation in MB-G4 (50 nM) under varying conditions: (**A**) increasing NMM concentrations (0.1, 0.5, 1, 2.5 μM) and (**B**) different KCl concentrations (0, 1, 5, 10, 50, 100 mM) at a fixed concentration of 0.5 μM of NMM.

Before the optimization of the experimental conditions with miR-155-3p, fluorescence studies to assess whether the G4 structure could be induced in the absence of the target were performed, with increasing concentrations of NMM and KCl (Figure 2). The ability of NMM to induce G4 formation was first evaluated by testing various concentrations (0.1, 0.5, 1, 2, and 5 μM) (Figure 2A). Fluorescence spectra analysis showed no significant differences between the conditions with and without MB-G4, suggesting that NMM alone did not induce G4 formation. However, at higher NMM concentrations, particularly at 5 μM, a second fluorescence peak at 609 nm started to appear while the initial peak at 621 nm remained present.

Subsequently, the effect of the KCl concentration (0, 1, 5, 10, 50, and 100 mM) was examined in the presence of 0.5 μM NMM (Figure 2B). At low KCl concentrations, fluorescence was primarily detected at 621 nm, but as the KCl concentration increased, a second peak at 609 nm appeared and gradually intensified. At 50 mM and 100 mM KCl, the 621 nm peak disappeared, and fluorescence was predominantly observed at 609 nm, indicating full G4 formation [24].

### 3.2. MB-G4 and miR-155-3p Interaction Studies

**Figure 3:**
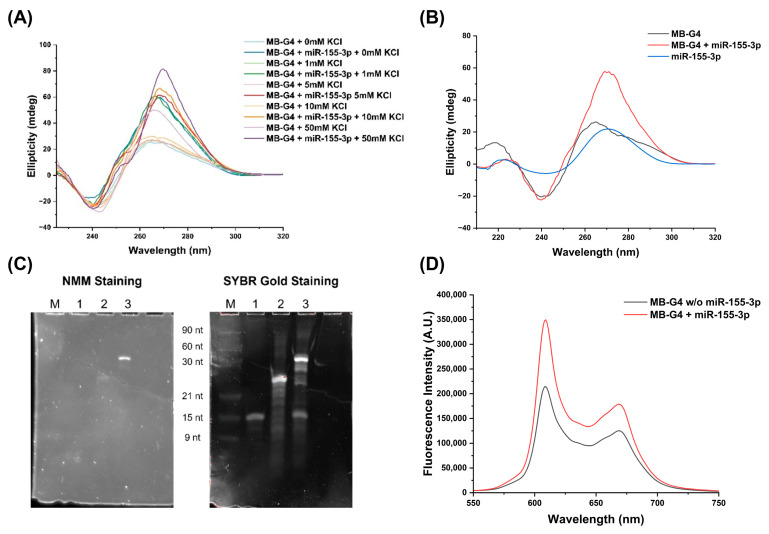
(**A**) CD spectra of MB-G4 at 5 μM in the presence and absence of miR-155-3p under increasing KCl concentrations (0, 1, 5, 10, 50 mM). (**B**) CD spectra under optimized conditions for MB-G4 (5 μM), miR-155-3p (2.5 μM), and MB-G4 + miR-155-3p (5 and 2.5 μM, respectively) for better visualization of structural differences. (**C**) Native PAGE analysis of miR-155-3p, MB-G4, and MB-G4 + miR-155-3p (all at 1 μM). (i) Post-staining with NMM; (ii) Post-staining with SYBR Gold. Lane M: Migration markers (90 nt, 60 nt, 30 nt, 21 nt, 15 nt, and 9 nt); Lane 1: miR-155-3p; Lane 2: MB-G4; Lane 3: MB-G4 + miR-155-3p. PAGE in the absence of KCl can be found in Figure S5. (**D**) Fluorescence spectra of NMM (1 μM) with MB-G4 (50 nM) alone and upon hybridization with miR-155-3p (25 nM).

CD spectroscopy was employed to investigate the structural transitions of MB-G4 under varying salt concentrations and upon hybridization with miR-155-3p (Figure 3A). The analysis focused on (i) the initial conformation of MB-G4, (ii) the effect of increasing KCl concentrations, and (iii) the structural changes upon target binding.

In the absence of miR-155-3p, MB-G4 exhibited a broad CD signal between 260 and 270 nm across all the salt concentrations tested. At 50 mM KCl, an increase in ellipticity with a peak tending toward 260 nm was observed. Upon the addition of miR-155-3p, a distinct shift in the positive CD peak to 267–270 nm was detected, alongside a general increase in ellipticity. The negative band around 240 nm remained present in all conditions.

Figure 3B presents the CD spectra of MB-G4, MB-G4 + miR-155-3p under optimized conditions (5 mM KCl), and miR-155-3p alone. The miR-155-3p spectrum displayed a peak at 270–275 nm. Upon hybridization with MB-G4, this peak became more pronounced, and the MB-G4 spectrum exhibited an ellipticity increase in the 260–270 nm region.

PAGE analysis was performed to further evaluate the structural changes upon miR-155-3p binding, and Figure 3C shows the gel stained sequentially with NMM and SYBR Gold in the presence of KCl. In Lane 2 (MB-G4 alone), a faint band was visible with NMM, indicating that a minor fraction of MB-G4 formed a G4 structure. In Lane 3 (MB-G4 + miR-155-3p), two distinct bands were detected with NMM staining, with the upper band showing stronger fluorescence. Subsequent staining with SYBR Gold revealed an additional band corresponding to unbound miR-155-3p which is also present in Lane 1 (miR-155-3p).

PAGE analysis was also conducted in the absence of KCl (Figure S5). Under these conditions, faint bands were detected with NMM staining in both the MB-G4 and MB-G4 + miR-155-3p samples.

Figure 3D displays the fluorescence spectra of MB-G4 in the presence and absence of miR-155-3p. The fluorescence intensity of MB-G4 alone was lower, while a significant fluorescence increase was observed upon target addition.

### 3.3. Specificity and Detection

**Figure 4:**
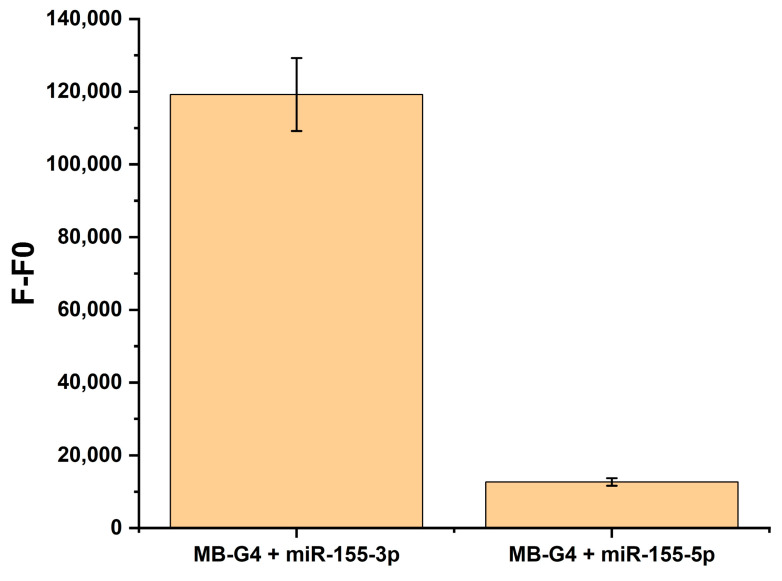
Specificity of MB-G4 in the presence of miR-155-3p and miR-155-5p under optimized conditions, where F represents the fluorescence intensity of MB-G4 in the presence of the respective microRNA, and F0 represents the fluorescence intensity of MB-G4 alone (without miRNA). The data shown are the differences between F and F0, reflecting the specific binding of MB-G4 to the target miRNA. Measurements were taken at 609 nm, data points represent the mean values of different measurements and are shown as mean ± SEM.

After optimizing the experimental conditions and confirming the conformational switch of MB-G4, detection studies were performed to evaluate its specificity for miR-155-3p (Figure 4). Given the importance of specificity in detection methods, miR-155-5p was used as a control. As shown in Figure 4, a pronounced fluorescence enhancement was observed in the presence of miR-155-3p compared to miR-155-5p. This result suggests that miR-155-3p effectively hybridized with the stem-loop structure of the MB-G4, releasing the G-rich sequence. Under the optimized conditions, the G-rich sequence formed a G4 structure that bound to NMM, leading to the observed fluorescence enhancement. In contrast, miR-155-5p did not induce a similar fluorescence response, indicating that it failed to hybridize with the MB-G4 or release the G-rich sequence necessary for G4 formation. These findings confirm the specificity of the MB-G4 for miR-155-3p.

**Figure 5:**
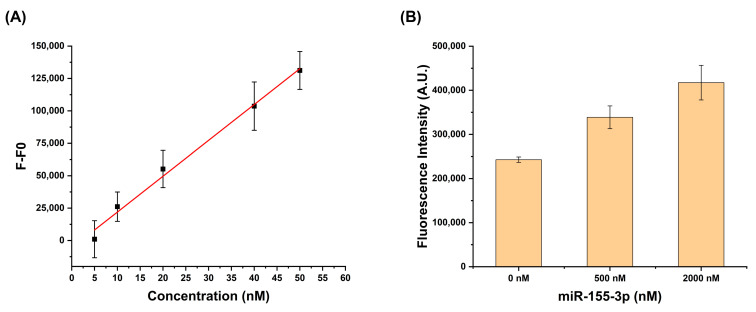
(**A**) Limit of detection (LOD) analysis of MB-G4 for miR-155-3p. A linear plot was obtained by measuring the fluorescence intensity as a function of increasing miR-155-3p concentrations (5, 10, 20, 40 50 nM). The trendline indicates the linear response range used to determine the LOD. (**B**) Detection of miR-155-3p in total RNA extracted from A549 cells. Fluorescence intensity was measured in the presence of total RNA to assess the performance of MB-G4 in a complex biological sample. All the data points were taken using fluorescence at 609 nm and represent the mean values of at least 3 different measurements and are shown as mean ± SEM.

The limit of detection (LOD) was determined by measuring fluorescence intensities at various miR-155-3p concentrations (Figure 5A). The LOD was found to be 10.85 nM, reflecting a balance among the fluorescence spectrometer’s sensitivity, background noise from the experimental setup, and G4 formation and stabilization efficiency. The linearity of the calibration curve was confirmed within the tested concentration range, with any deviations attributed to saturation effects or baseline noise. The LOD was influenced by the optimized experimental conditions (e.g., 5 mM KCl, 10 mM Licaco, and 1 µM NMM), which helped reduce nonspecific fluorescence.

Studies using the total RNA extracted from the lung cancer cell line A549 were performed to evaluate the MB-G4 detection system’s ability to selectively detect miR-155-3p in a complex RNA environment (Figure 5B), where interference from other RNA species could occur. For this, 250 ng of total RNA was spiked with different concentrations of synthetic miR-155-3p, and the results were compared with those from MB-G4 samples containing total RNA without spiking. The results showed that the MB-G4 successfully detected its target even in the presence of a diverse range of RNAs, demonstrating the reliability and specificity of the method.

## 4. Discussion

For the assessment of G4 formation by MB-G4 in the absence of the target, the results suggest that NMM (Figure 2A) alone does not strongly induce G4 formation but can interact with the MB-G4 structure at high concentrations, leading to minor spectral changes. With increasing KCl concentration (Figure 2B), a gradual emergence of a new peak is observed at 609 nm and eventually becomes dominant over the 621 nm peak. This transition suggests a stable G4 conformation, as the only varying condition is the salt concentration, which is crucial for G4 stabilization because, at the lowest KCl concentration, only a single peak is observed, whereas at higher concentrations, the 621 nm peak fully disappears, and the 609 nm peak increases and sharpens (features consistent with G4 formation and NMM interaction) [25]. Furthermore, the 609 nm peak is widely recognized in NMM-G4 experiments, corroborating its role as an indicator of G4 presence and stabilization. This behavior aligns with the well-documented role of KCl in stabilizing G4 structures through coordination with guanine quartets, as well as a potential synergistic effect between high KCl concentrations and the G4 ligand, both of which can independently stabilize the G4 structure [26].

To minimize unintended G4 formation while ensuring structural stability in the presence of miR-155-3p, 5 mM KCl was selected as the optimal condition, since at this concentration, both fluorescence peaks were present, suggesting an intermediate state where the G4 structure is not fully stabilized. This ensures that complete G4 formation—and the corresponding fluorescence change—only occurs upon miR-155-3p binding. Higher KCl concentrations (50 mM and 100 mM) were excluded, as they led to a full stabilization of the G4 structure without the target, potentially resulting in false-positive signals.

From the interaction studies between miR-155-3p and MB-G4, the CD spectra indicate that MB-G4 exists in a dynamic equilibrium between its hairpin conformation and a partially accessible G-rich sequence capable of forming G4 structures. A B-form-like conformation was dominant, characterized by a long positive wavelength between 260 and 280 nm and a negative peak around 240 nm, consistent with the stem-loop structure [27,28]. The emergence of a peak at 260 nm at 50 mM KCl suggests a partial destabilization of the molecular beacon, allowing some G4 formation [29]. Upon the addition of miR-155-3p, a significant increase in ellipticity was observed in all the conditions, with a deviation in the positive CD peak to 267–270 nm. This shift is characteristic of RNA–DNA hybrid duplex formation, which typically adopts an A-form-like conformation [30]. The increased signal intensity further supports successful hybridization, stabilizing the MB-G4 in an open conformation [27]. Notably, this shift contrasts with the peak at 260 nm observed under 50 mM KCl without the target, reinforcing that the dominant structural change upon target binding is the formation of a stable hybridized duplex rather than a G4-dominated structure.

Additionally, negative bands around 240 nm were observed in all conditions. These bands are typical of the DNA/RNA backbone and support the A-form duplex structure formed upon hybridization with miR-155-3p, but they are also common for G4 structures and/or the stem-loop originating from the hairpin structure [29,30].

Figure 3B presents the CD spectra of MB-G4, MB-G4 + miR-155-3p under optimized conditions (5 mM KCl), and miR-155-3p alone for comparison. The miR-155-3p spectrum exhibits a distinct peak around 270–275 nm, characteristic of single-stranded RNA [31]. Upon hybridization with MB-G4, this peak becomes more pronounced, indicating the formation of a DNA–RNA hybrid. The MB-G4 spectrum remains consistent with the previous observations, with increased ellipticity in the 260–270 nm region upon target binding. This supports that the structural transition upon miR-155-3p binding is primarily due to hybridization, which facilitates the release of the G-rich sequence, allowing for subsequent G4 formation.

Although the analysis focuses more on the hybridization suggested by the CD signals, this does not entirely exclude the possibility of G4 formation. Once hybridization occurs, the G4 structure remains free to form under the right salt concentrations. Thus, while hybridization leads to the formation of the RNA–DNA duplex, G4 formation can still coexist under favorable conditions.

The PAGE results further confirm these structural transitions. The presence of a weakly fluorescent band in MB-G4 alone (Lane 2, Figure 3C) suggests that a minor fraction of the beacon can form a G4 without the target. However, the strong fluorescence of the upper band in Lane 3 confirms that miR-155-3p binding facilitates the conformational switch, exposing the G-rich sequence for stable G4 formation. As for Lane 1, corresponding to miR-155-3p, no signal was observed when stained with SYBR Gold, reinforcing the specificity of NMM for G4s. Furthermore, when stained with SYBR Gold, an additional band was observed in Lane 3, corresponding to the unbound target, and the multiple bands observed for MB-G4 could be attributed to the various isoforms that MB-G4 can adopt, none of which form a G4 structure, as evidenced by the lack of detection with NMM staining. The PAGE results in the absence of KCl (Figure S5) suggest that some G4 structures were formed to a limited extent, possibly due to the ability of NMM to stabilize G4 structures. However, the fluorescence intensity of these bands was significantly lower compared to the intense band observed in the presence of 5 mM KCl. These results underscore the critical role of the target miR-155-3p and KCl in facilitating and stabilizing efficient G4 formation within the MB-G4 system.

The above findings align with the well-established role of KCl in stabilizing G4 structures. The gradual disappearance of the 621 nm fluorescence peak with increasing KCl concentrations, along with the emergence of the 609 nm peak, suggests a transition toward a stable G4 conformation in the G4 assessment studies. While hybridization primarily results in an RNA–DNA duplex, the released G-rich sequence remains capable of forming a G4 structure, provided that the salt conditions are favorable, as confirmed by the interaction studies.

The fluorescence results (Figure 3D) reinforce these observations. The lower fluorescence of MB-G4 alone suggests that the G-rich sequence was sequestered, preventing significant NMM binding. The strong fluorescence upon miR-155-3p addition confirms that the target promoted the structural transition necessary for effective NMM recognition. However, the observed fluorescence was higher than expected, likely due to the fraction of the MB-G4 not fully adopting the stem-loop conformation, thereby allowing some G4 formation, as observed in CD and PAGE analysis, as well as subsequent recognition by NMM, resulting in fluorescence emission.

The results regarding the specificity of the method (Figure 4) confirm that the MB-G4 exhibits high specificity for miR-155-3p. The pronounced fluorescence enhancement observed in the presence of miR-155-3p compared to miR-155-5p demonstrates that the MB-G4 effectively recognizes its target and undergoes the conformational change necessary for G4 formation. The lack of fluorescence enhancement in the presence of miR-155-5p further supports the specificity of this approach.

The determination of the LOD as 10.85 nM highlights the sensitivity of the MB-G4 detection system, as it balances fluorescence spectrometer sensitivity with the potential for background interference. The linear calibration curve within the tested range further strengthens the reliability of the assay. The optimized experimental conditions, such as the specific concentrations of KCl, Licaco, and NMM, were crucial in minimizing nonspecific fluorescence and improving the overall detection sensitivity. Table 2 presents a comparison between our method and the other methods for the detection of miRNAs, including miR-155-3p. Although the LOD value found for this work is higher than some of the methods already employed, it must be kept in mind that MB-G4’s ability to perform rapid, label-free detection makes it a cost-effective alternative to other molecular beacon-based methods, which often require more complex and costly modifications. The conformational changes inherent to its design provide an innovative and efficient approach to target detection, further emphasizing the potential of MB-G4 for practical applications in molecular diagnostics.

Additionally, the ability of MB-G4 to selectively detect miR-155-3p in complex RNA environments (Figure 5B), such as total RNA extracted from lung cancer cells, is a significant finding. The successful detection of miR-155-3p in the presence of other RNA species demonstrates that the MB-G4 system is robust and reliable, even under challenging conditions. This suggests that MB-G4 is well-suited for use in complex biological samples, offering a promising method for target detection without the need for expensive chemical modifications.

## 5. Conclusions

In conclusion, the MB-G4 system introduces a rapid, enzyme-free, and precise method for detecting miR-155-3p, highlighting a different application of G4 structures beyond their traditional roles in DNA/RNA regulation. Through the optimization of experimental conditions, including salt concentration, ligand concentration, and hybridization temperature, we established a reliable platform for miRNA detection. Fluorescence and CD spectroscopy confirmed the conformational changes induced by miR-155-3p binding, leading to G4 formation and subsequent fluorescence enhancement. The specificity of MB-G4 was validated through comparisons with a control miRNA (miR-155-5p), its limit of detection (LOD) with miR-155-3p was determined to be 10.85 nM, and its applicability in complex biological samples was demonstrated with the total RNA extracted from A549 cells, indicating its high sensitivity and selectivity for its target. While G4-based detection is not typically the most straightforward method for target identification, the MB-G4 system showcases the unique conformational dynamics of G4s in response to miRNA hybridization, offering a distinct approach to molecular detection. By leveraging G4s’ ability to undergo structural transitions and bind specific ligands like NMM, this method provides a cost-effective, label-free solution for miRNA analysis, particularly in the context of miRNA-based biomarkers in diseases like cancer. Despite challenges in G4-based detection, this study demonstrates how G4s can be effectively utilized for innovative molecular diagnostics.

## Figures and Tables

**Table 1:** Oligonucleotide sequences used in this work.

Sequence Name	Sequence (5′→ 3′)	ntNumber	Ɛ(L·mol^−1^·cm^−1^) at 260 nm
Molecular Beacon(MB-G4)	TACCCTACTGTTAATGCTAATATGTAGGAGACTGATTGGGTAGGGTAGGGTAGGG	55	553,900
miR-155-3p	CUCCUACAUAUUAGCAUUAACA	22	206,500
miR-155-5p	TTAATGCTAATCGTGATAGGGGT	23	230,300

Red: miR-155-3p recognition region; Green: G-rich sequence prone to G4 formation.

**Table 2:** Comparison of current method with other methods to detect miRNAs.

Detection Method	LOD	Target	Ref.
Colorimetric Detection with Gold Nanoparticles	100 aM	miRNA-10b	[32]
Gold and silver nanorod/thionine/complementary DNA composite	1 pM	miRNA-155	[33]
Fluorophore and quencher dyes conjugated molecular beacon	0.66 nM	miR-155-3p	[28]
Molecular Beacon Bead-Based	42 nM	miR-155-3p	[34]
MB-G4	10.85 nM	miR-155-3p	This work

## Data Availability

The original contributions presented in this study are included in the article/Supplementary Materials. Further inquiries can be directed to the corresponding author.

## Supplementary Materials

The following supporting information can be downloaded at: https://www.mdpi.com/article/10.3390/biom15030410/s1, Figure S1: Optimization of KCl concentration for MB-G4 in the presence of miR-155-3p; Figure S2: Optimization of NMM concentration for MB-G4 in the presence of miR-155-3p; Figure S3: Optimization of temperature for MB-G4 in the presence of miR-155-3p; Figure S4: Optimization of hybridization time for MB-G4 with miR-155-3p; Figure S5: PAGE under native conditions results of miR-155-3p, MB-G4, and MB-G4 + miR-155-3p at 1 μM; Table S1: Molecules used in this study.
