# Supplementary material for: G-Quadruplex Conformational Switching for miR-155-3p Detection Using a Ligand-Based Fluorescence Approach
Source: Biomolecules. 2025 Mar 13;15(3):410. doi: 10.3390/biom15030410 (PMC11940483; doi:10.3390/biom15030410)
Supplement: Supplementary file 1 [file biomolecules-15-00410-s001.zip › biomolecules-3510926-supplementary.pdf]

Supplementary Materials

Table S1. Molecules used in this study.

Name	Chemical Structure	Molecular Weight (g.mol^{-1})	CAS number Reference
**NMM** (N-methyl mesoporphyrin IX)	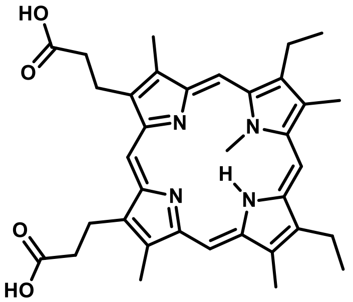	580.7	142234-85-3
**NMM**: 8,13-diethyl-3,7,12,17,23-pentamethyl-21H,23H-porphine-2,18-dipropanoic acid			

Optimization of experimental conditions with miR-155-3p

After confirming that the selected concentrations of KCl and NMM do not induce G4 formation in the absence of the target, we proceeded to optimize the experimental conditions in the presence of miR-155-3p. MB-G4 was annealed at 95ºC for 5mins and then immersed in ice for 10mins prior to the studies. The concentrations of MB-G4 and miR-155-3p were maintained at 50 nM and 25 nM, respectively, in a buffer containing 10 mM LiCaco and 10 mM $MgCl_2$.

KCl Optimization

Varying KCl concentrations (0 mM, 1 mM, 5 mM, 10 mM, 50 mM, and 100 mM) were tested in the presence of 0.5 μM NMM. Samples were heated at 40°C for 60 minutes prior to fluorescence analysis.

The resulting spectra showed similar trends to those observed in the absence of the target at the lowest salt concentrations (0 mM and 1 mM). At these concentrations, no peak at 609 nm was detected, and no fluorescence enhancement occurred, indicating that these salt levels were insufficient for G4 formation, even in the presence of the target. At 1 mM KCl, a peak began to appear at 609 nm, while at 0 mM, the broad, low-intensity peak remained centered at 621 nm.

In contrast, for KCl concentrations of 5 mM and 10 mM, there was a significant increase in fluorescence intensity and a clear peak at 609 nm. This suggests that these salt concentrations are adequate to stabilize the G4 structure when miR-155-3p binds to the MB-G4. At higher salt concentrations (50 mM and 100 mM), the spectra displayed similar behavior, with a sharp, prominent peak at 609 nm. However, this fluorescence enhancement was attributed not only to target-induced G4 formation but also to G4 stabilization facilitated by KCl alone.

Therefore, the results indicate that 5 mM and 10 mM KCl provide optimal conditions for selective G4 stabilization upon target binding, while minimizing non-specific G4 formation induced by high salt concentrations, and since the minimum KCl-induced G4 formation was a priority, subsequent studies were carried out under conditions with 5 mM KCl.

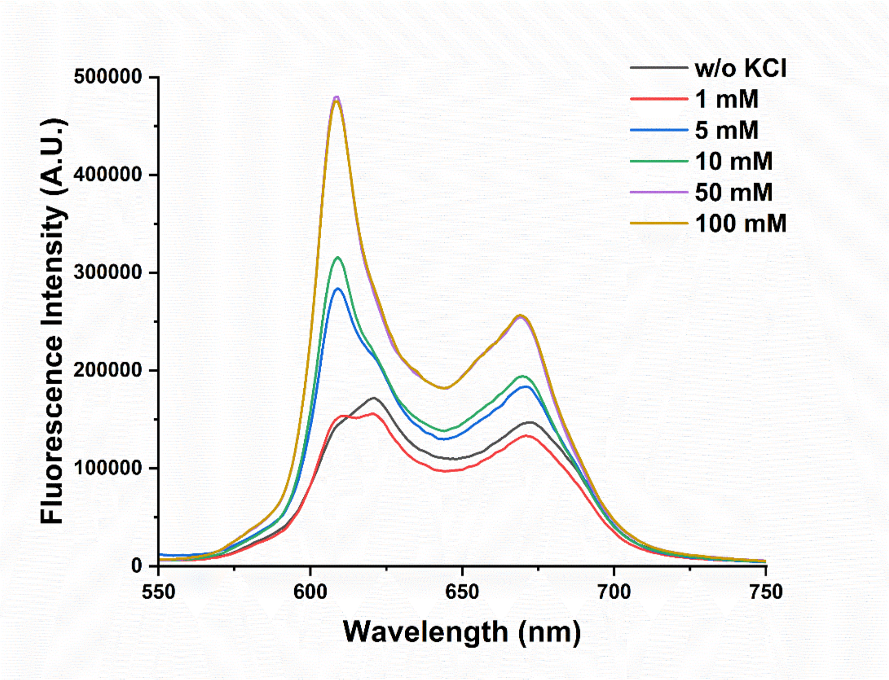

Figure S1. Optimization of KCl concentration for MB-G4 in the presence of miR-155-3p. Assays were performed in duplicate.

NMM Optimization

To determine the optimal concentration of NMM, its levels were varied (0 µM, 0.1 µM, 0.5 µM, 1 µM, 2 µM, and 5 µM) in a buffer containing 5 mM KCl, 10 mM LiCaco, and 10 mM MgCl2.

The results showed that NMM exhibited lower fluorescence emission in the absence of the target, while a significant enhancement in fluorescence was observed upon the addition of the target. This enhancement was accompanied by a noticeable increasing of the peak at 609 nm as well as the sharpness of the fluorescence signal.

Based on these findings, a concentration of 1 µM NMM was selected for subsequent studies, as it produced the most pronounced changes in peak intensity and sharpness.

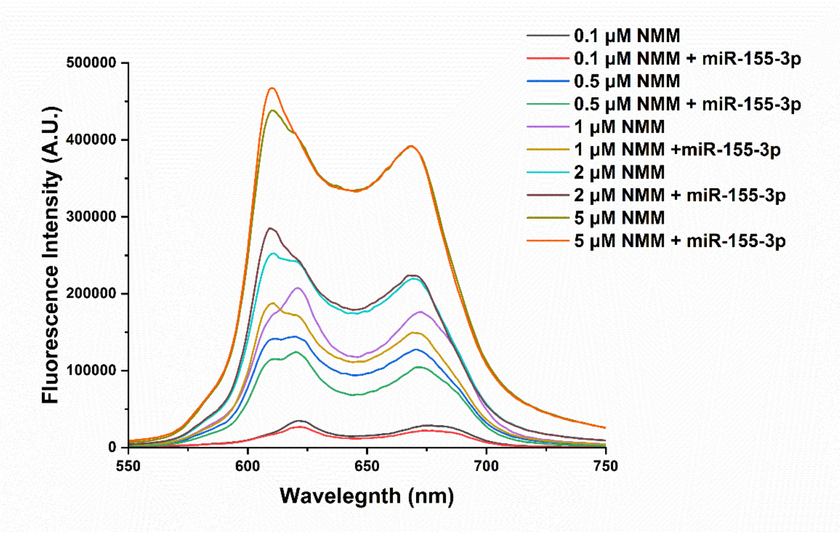

Figure S2. Optimization of NMM concentration for MB-G4 in the presence of miR-155-3p. Assays were performed in duplicate.

Temperature Optimization

The effects of hybridization temperature on the interaction between miR-155-3p and the MB-G4 were assessed by testing a range of temperatures (25°C, 30°C, 40°C, 50°C, 60°C, and 70°C) for a duration of 60 minutes, followed by fluorescence analysis.

At the lowest temperatures (25°C and 30°C), fluorescence differences were minimal. This outcome aligns with expectations, as hybridization of short nucleic acid sequences typically requires higher temperatures, approximately 50–60°C, for optimal efficiency. In contrast, temperatures of 40°C, 50°C, and 60°C produced significantly higher fluorescence signals, indicating enhanced hybridization and subsequent G4 formation. However, at 60°C and 70°C, a reduction in fluorescence intensity was observed, suggesting that these elevated temperatures might destabilize the hybridization process.

Moreover, significant peak emergences were observed across the conditions. In the presence of the target, a distinct more pronounced peak at 609 nm was observed, whereas in its absence, the double broad peak characteristic of unbound MB-G4 was maintained. These observations strongly support the hypothesis that the observed spectral changes are directly linked to target hybridization and G4 formation.

The highest optimal temperature was 50 °C and therefore chosen for future studies.

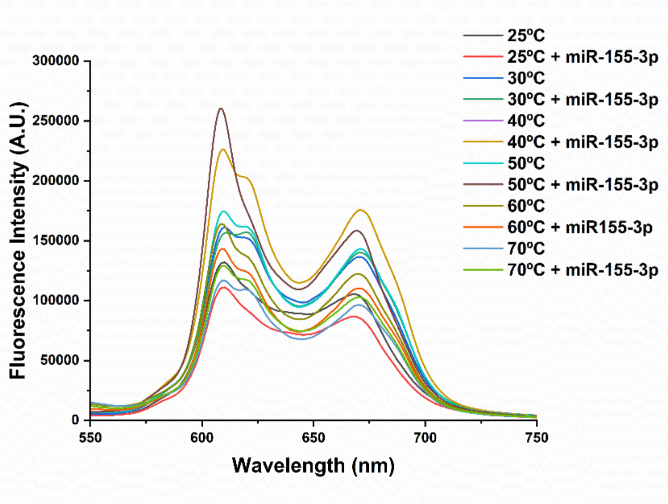

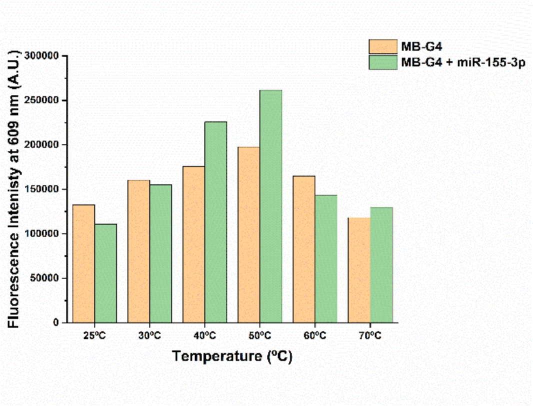

Figure S3. Optimization of temperature for MB-G4 in the presence of miR-155-3p. Assays were performed in duplicate.

Time Optimization

The primary goal of this study is to develop a rapid, enzyme-free detection method for miR-155-3p. A key aspect of this approach is determining the minimum incubation time required for effective hybridization between the target miRNA and the MB-G4.

To identify the optimal hybridization time, a series of time intervals (0, 10, 20, 30, 40, 50, and 60 minutes) were systematically evaluated under controlled conditions and fluorescence measurements were taken for samples containing only the MB-G4 and those containing both the MB-G4 and the target miR-155-3p. The results showed that fluorescence intensity reached a stable plateau after 30 minutes, regardless of the presence of the target, indicating that hybridization between the MB-G4 and miR-155-3p, along with any related conformational changes, is completed effectively within this time frame.

Based on these findings, an incubation time of 30 minutes was selected for subsequent experiments. This duration provides a balance between rapid detection and adequate time for reliable hybridization, aligning with the goal of creating a time-efficient detection method for miR-155-3p.

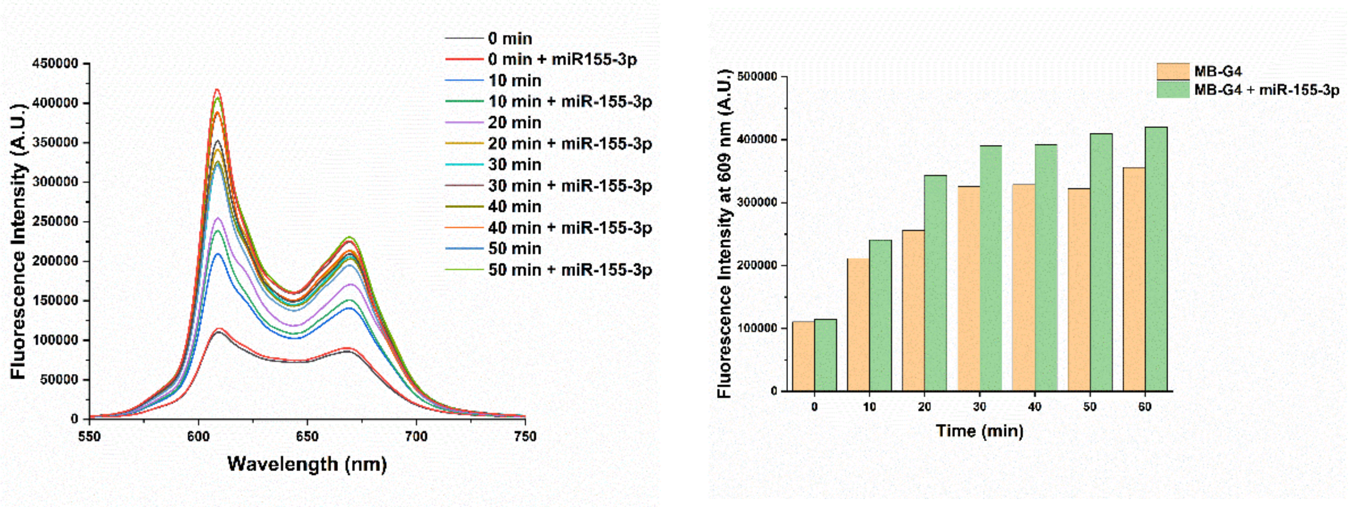

Figure S4. Optimization of hybridization time for MB-G4 with miR-155-3p. Assays were performed in duplicate.

PAGE

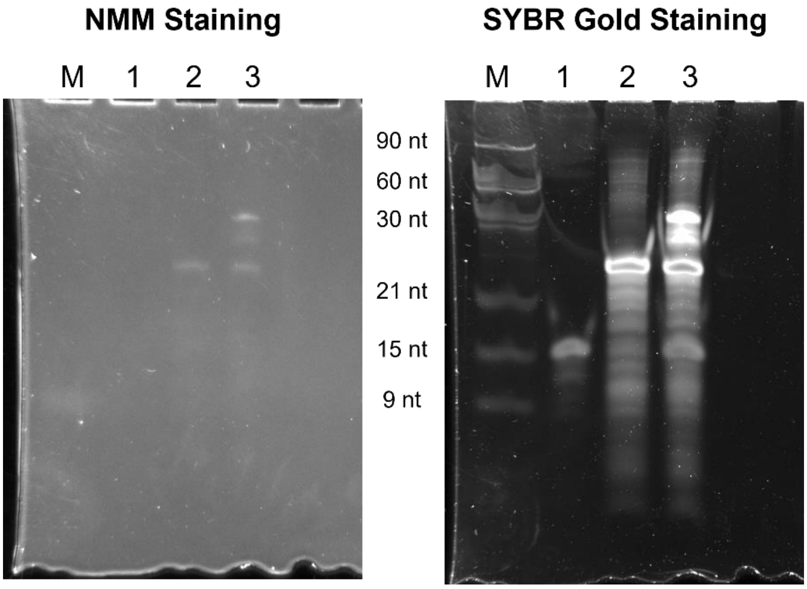

Figure S5. PAGE under native conditions results of miR-155-3p, MB-G4, and MB-G4 + miR-155-3p at 1 μM. (i) Post-stain with NMM; (ii) Post-stain with SYBR Gold. Lane M: Migration markers 90 nt; 60 nt; 30 nt, 21 nt, 15 nt and 9 nt; Lane 1: miR-155-3p, Lane 2: MB-G4 and Lane 3: MB-G4 + miR-155-3p.
